# Delivery of Circular RNAs into Splenic Immune Cells via Intravenous Administration of Polyaspartamide Derivative Polyplexes

**DOI:** 10.1021/acsbiomaterials.5c02147

**Published:** 2026-03-27

**Authors:** Jun Su An, Sung Been Lim, Kyung Hyun Lee, Seongcheol Kim, SeonJeong Kim, Jieun Lee, Dongsu Kim, Seung Ryul Han, Mitsuru Naito, Kanjiro Miyata, Hyun Jin Kim, Seong-Wook Lee

**Affiliations:** † Department of Biological Sciences and Bioengineering, 105904 Inha University, 100 Inha-ro, Michuhol-gu, Incheon 22212, Republic of Korea; ‡ R&D Center, 661262 Rznomics Inc., Seongnam 13486, Republic of Korea; § Department of Materials Science and Technology, Faculty of Advanced Engineering, Tokyo University of Science, 6-3-1 Niijuku, Katsushika-ku, Tokyo 125-8585, Japan; ∥ Center for Advanced Modalities and DDS, The University of Osaka, 1-10 Yamadaoka, Suita-shi, Osaka 565-0871, Japan; ⊥ Department of Materials Engineering, Graduate School of Engineering, The University of Tokyo, 7-3-1 Hongo, Bunkyo-ku, Tokyo 113-8656, Japan; # Department of Medicinal Biosciences and Bioengineering, Inha University, 100 Inha-ro, Michuhol-gu, Incheon 22212, Republic of Korea; ∇ Department of Biological Engineering, College of Engineering, Inha University, 100 Inha-ro, Michuhol-gu, Incheon 22212, Republic of Korea; ○ Biohybrid Systems Research Center, Inha University, 100 Inha-ro, Michuhol-gu, Incheon 22212, Republic of Korea; ◆ Department of Bioconvergence Engineering, Research Institute of Advanced Omics, 34937 Dankook University, Yongin 16890, Republic of Korea

**Keywords:** circular RNA, polyplex, cationic polymer, spleen

## Abstract

*In vivo* chimeric antigen receptor (CAR) cell engineering, such as CAR T, induces CAR expression in target immune cells via the systemic administration of CAR constructs. This study investigated polyplex formation with circular RNAs to evaluate its systemic delivery efficacy into the spleen, where an abundance of various immune cells resides. Firefly luciferase (FLuc)-coded circular RNAs with Coxsackievirus B3 internal ribosome entry site (IRES) were synthesized via end-to-end self-targeting and splicing reaction. A cationic polyaspartamide derivative, PAsp­(DET/CHE), which enhances endosomal escapability and optimizes hydrophobicity for particle stability, has been utilized to formulate linear or circular RNAs. PAsp­(DET/CHE) successfully formulated circular RNAs into polyplexes with physicochemical characteristics similar to linear RNA polyplexes, and their polyplexes significantly improved RNA delivery efficacy in various cultured immune cells. PAsp­(DET/CHE) polyplexes achieved spleen-targeted delivery of linear and circular RNAs after intravenous administration, regardless of RNA payloads, and induced high FLuc expression for up to 48 h. Notably, the circular RNA-loaded polyplexes exhibited higher T cell delivery efficacy with relatively lower accumulation in dendritic cells and macrophages than the linear RNA-loaded polyplexes. Therefore, the optimized polyplex formulation with circular RNAs can be utilized as an *in vivo* CAR RNA delivery platform for future T cell therapies.

## Graphical abstract

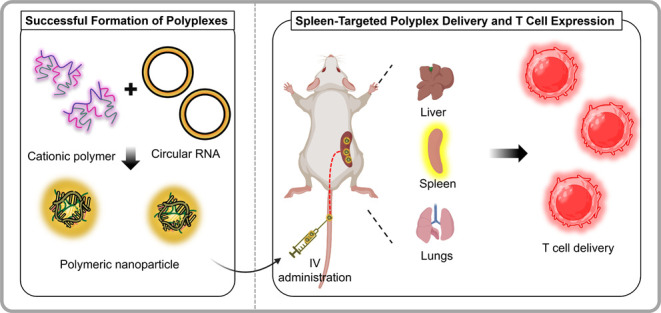

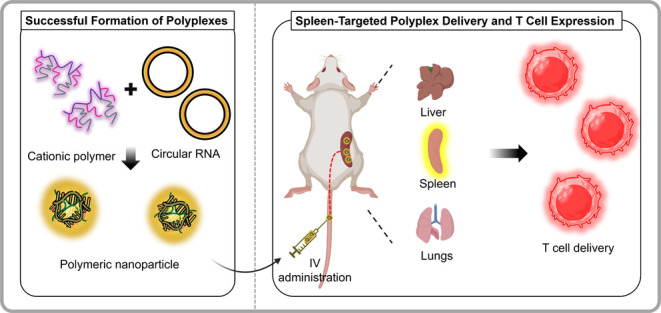

## 1 Introduction

The remarkable clinical activity of chimeric antigen receptor (CAR) T therapies in B cell and plasma cell malignancies has validated the use of this therapeutic class for liquid cancers.^1,2^ CARs are synthetic modular proteins that redirect immune cell reactivity toward a target of interest. The production of CAR T cells relies on complex and expensive processes of *ex vivo* cell engineering. Briefly, patient T cells are isolated, modified using viral vectors to express CAR proteins, expanded, and reinfused.^3,4^ Manufacturing processes are performed manually or in a semiautomated manner, which contributes to product variability and is expensive.^4^ Thus, there is a need for new approaches to engineer CAR T cells that can kill cancer cells while reducing the disadvantages of *ex vivo* cell engineering.

*In vivo* CAR cell engineering induces the expression of CAR molecules in immune cells, including T cells, via systemic administration of CAR constructs.^5–7^ Recent nonviral vectors, such as lipid nanoparticles (LNPs) and polymeric nanoparticles (PNPs), can specifically deliver mRNA into the spleen via intravenous administration. This is achieved using LNPs with optimized chemical structures of lipid components or modification of antibody conjugation.^8–10^ Indeed, mRNA avoids the safety issues associated with viral vectors, such as insertional mutagenesis and *in vivo* immunogenicity.^11^ Additionally, mRNA induces transient CAR expression, which reduces the risks associated with the long-term activity of CAR-expressing immune cells. Furthermore, mRNA-based *in vivo* CAR cell engineering permits repeated dosing with effective CAR constructs, broadening the possibility of clinical translation.^12^ An amphiphilic polyaspartamide derivative, PAsp­(DET/CHE), which contains diethylenetriamine (DET) for endosomal escape ability and mRNA binding, and 2-cyclohexylethylamine (CHE) for enhanced nanoparticle stability, efficiently expressed mRNA in various organs such as the lung and muscle via systemic and local administration.^13–15^ Indeed, spleen-specific mRNA expression was achieved by adjusting the physicochemical properties of the PAsp­(DET/CHE) polyplexes, such as surface charge and particle size.^16^

Circular RNA, a covalently closed loop of RNA, has a unique ring-like endless structure that renders it resistant to degradation by exonucleases, thereby enhancing its shelf life and biological stability.^17^ Circular RNA exhibits a significantly extended *in vivo* half-life, up to 2.5-fold longer than linear mRNA.^18^ Unlike linear mRNA, circular RNA does not require 5′ cap analogs, modified nucleotides such as N1-methyl-pseudouridine (m1ψ),^19^ or a 3′ poly­(A) tail for translation and stability. Instead, translation is initiated in a cap-independent manner via an internal ribosome entry site (IRES), which was first discovered in RNA viral genomes^20^ and is now the standard mechanism for circular RNA translation.^21^ Circular RNA has been utilized in LNP platforms to induce high levels of therapeutic proteins in the treatment of various diseases, such as lung cancer^22^ and ocular neurodegeneration.^23^ However, no studies have explored the efficacy of circular RNA delivery to the spleen using cationic polymers.

In the present study, we investigated the delivery efficacy of circular RNA into splenic immune cells using PAsp­(DET/CHE) for *in vivo* CAR cell engineering (Scheme 1A,1B). Firefly luciferase (FLuc)-coded circular RNAs with Coxsackievirus B3 (CVB3) IRES were prepared via end-to-end self-targeting and splicing (STS) reactions using *Tetrahymena* group I intron ribozyme,^24^ which has the advantage of leaving no intronic scars in the generated circular RNAs (Scheme 1C), compared to conventional ribozyme-based self-circularization methods such as permuted intron-exon (PIE).^25^ Unlike FLuc linear RNAs (or FLuc mRNAs), which require modified nucleotides (5-methoxyuridine [5 moU] in this study) for efficient translation to avoid unwanted innate immunity,^26^ CVB3 IRES-based FLuc circular RNAs were prepared using only natural nucleotides. PAsp­(DET/CHE) polyplexes containing FLuc linear or circular RNA were prepared at a predetermined molar ratio of amino groups in the polymer to phosphate groups in the RNA (*N*/*P*). Polyplexes at the *N*/*P* exhibited efficient delivery of mRNA into the spleen via intravenous administration in the previous study.^16^ Linear and circular RNA-loaded polyplexes were systematically compared in terms of their physicochemical characteristics, RNA transfection into immune cells, and cellular internalization. Finally, both polyplexes were evaluated for their biodistribution and accumulation in splenic immune cells after systemic administration to determine their therapeutic potential in T-cell-based cancer immunotherapy.

**Scheme 1:**
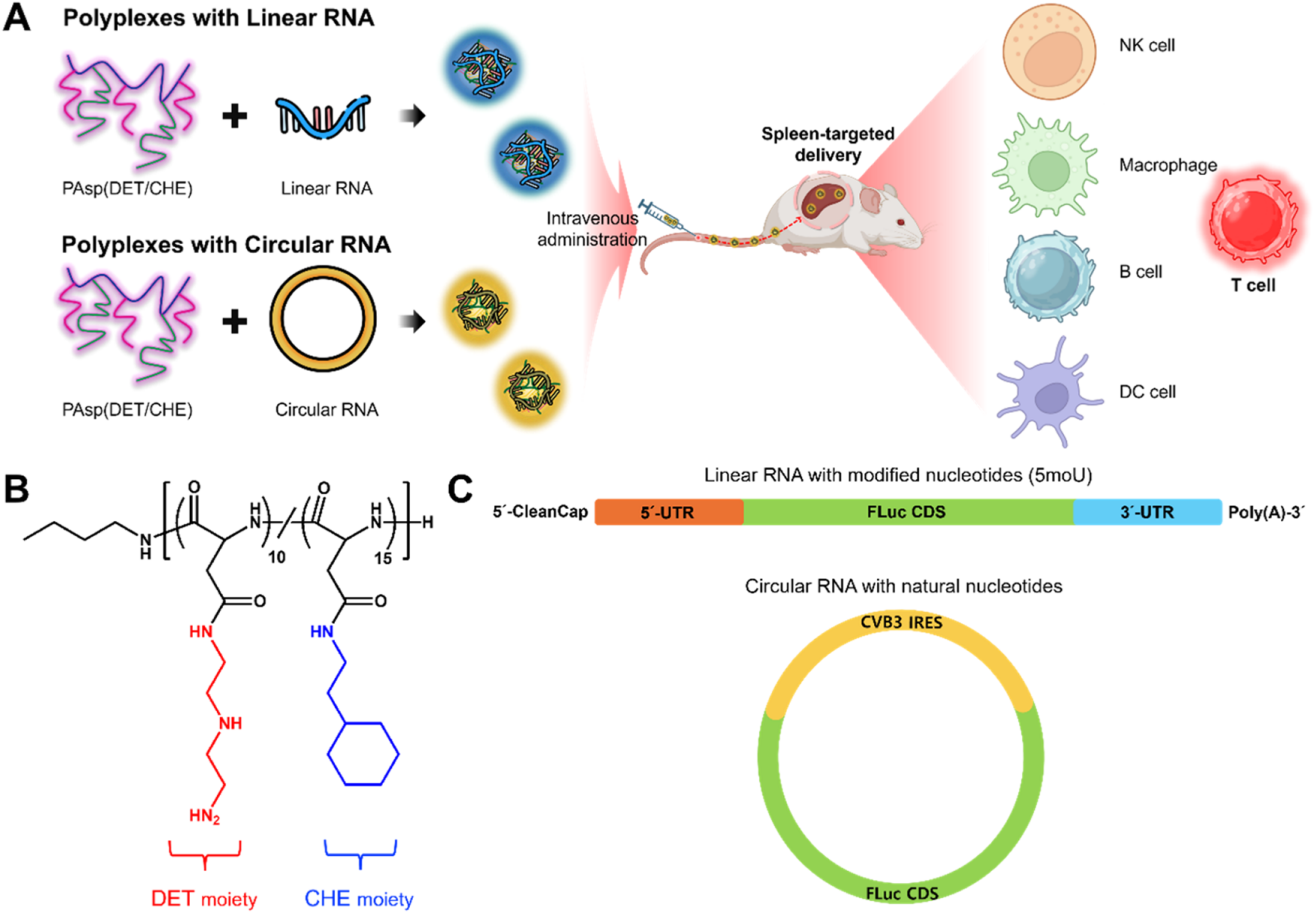
(A) Schematic Illustration of the Present Study; (B) Chemical Structure of PAsp­(DET/CHE); and (C) Structures of FLuc Linear and Circular RNAs

## 2 Materials and Methods

### 2.1 Materials

Dimethylformamide (DMF) was purchased from Fisher Scientific (Waltham, MA, USA). Dichloromethane (DCM), hexane, ethyl acetate, *n*-butylamine, diethylenetriamine (DET), *N*-methyl-2-pyrrolidone (NMP), methanol (MeOH), triethylamine (TEA), and Nile red (NR) were purchased from Sigma-Aldrich (St. Louis, MO, USA). DCM, *n*-butylamine, DET, and NMP were distilled for polymerization and aminolysis. Hydrochloric acid (HCl) was purchased from J. T. Baker (Phillipsburg, NJ, USA). Diethyl ether was purchased from DAEJUNG CHEMICALS & METALS CO., Ltd. (Siheung-si, Gyeonggi-do, Republic of Korea). β-Benzyl-l-aspartic acid *N*-carboxyanhydride (BLA-NCA) was purchased from Chuo Kaseihin (Tokyo, Japan). Roswell Park Memorial Institute 1640 (RPMI 1640), Minimum Essential Medium α (MEMα), l-glutamine, Dulbecco’s phosphate-buffered saline (D-PBS(−)), horse serum (HS), penicillin–streptomycin (PS, 10,000 U/mL), and trypsin-ethylenediaminetetraacetic acid (trypsin-EDTA) were purchased from Gibco (Waltham, MA, USA). 2-Cyclohexylethylamine (CHE) was purchased from Tokyo Chemical Industry Co., Ltd. (Tokyo, Japan). HEPES buffer (1 M, pH 7.3) was purchased from Amresco (Solon, OH, USA). Fetal bovine serum (FBS) was purchased from Invitrogen (Waltham, MA, USA). Cell scrapers were purchased from SPL (Pocheon-si, Gyeonggi-do, Republic of Korea). Ultrapure agarose was purchased from Thermo Fisher Scientific (Waltham, MA, USA). VivoGlo Luciferin was purchased from Promega (*in vivo* grade, P1043). Human natural killer NK-92 mi and human T lymphoblast Jurkat cells were obtained from the American Type Culture Collection (Manassas, VA, USA). RAW 264.7 cells, murine monocyte/macrophage cell lines, were received from the Korean Cell Line Bank (Seoul, Republic of Korea). Firefly luciferase-coded mRNA with 5′-CleanCap, 5 moU, and 3′-polyA (FLuc mRNA, L-7202) was purchased from TriLink Biotechnologies (San Diego, CA, USA). Fluorescently labeled FLuc linear and circular RNAs were prepared by attaching fluorescent dyes using a Label IT Cy5 labeling kit (Mirus Bio Corporation, Madison, WI, USA). ^1^H NMR was analyzed by Avance III (400 MHz, Bruker, Billerica, MA, USA).

### 2.2 Cell Culture

Jurkat cells were cultured in RPMI 1640 supplemented with 10% FBS and 1% PS at 37 °C in a humidified incubator containing 5% CO_2_. The cells were routinely maintained by adding fresh medium every two days. NK-92 mi cells were cultured in MEMα supplemented with 10% FBS, 10% HS, 1% PS, and 2 mM l-glutamine. RAW 264.7 cells were cultured in RPMI 1640 supplemented with 10% FBS and 1% PS. The cells were gently detached using a cell scraper every 3 days, collected by centrifugation at 1,000 rpm for 5 min, and resuspended in fresh culture medium.

### 2.3 Synthesis of Poly­(β-benzyl-l-aspartate) (PBLA)

PBLA was synthesized via ring-opening polymerization of BLA-NCA as a monomer initiated by *n*-butylamine, as described previously.^27,28^ *n*-Butylamine (3.20 μL, 0.032 μmol) was directly added to BLA-NCA (200 mg, 0.81 mmol) dissolved in DCM/DMF (3 mL, 9:1 v/v). The polymerization was performed at 37 °C for 72 h under an argon atmosphere. The polymer was purified via precipitation in hexane/ethyl acetate (6:4 v/v) and dried overnight under reduced pressure. Polymers were characterized by ^1^H NMR (400 MHz, Avance III, Bruker, Billerica, MA, USA) and gel permeation chromatography (GPC). The GPC system (HLC-8420, TOSOH CORPORATION, Tokyo, Japan) equipped with two TSK gel columns (G3000HHR and G4000HHR) was eluted with DMF containing lithium chloride (10 mM) at 1.0 mL/min. The degree of polymerization (DP) of the BLA unit in PBLA was calculated to be 25 from the peak intensity ratio of the phenyl protons (COOCH_2_C_6_H_5_, δ = 7.18–7.40) to the methyl protons (CH_3_(CH_2_)_3_NH, δ = 0.8) in the ^1^H NMR spectrum (Figure S1A). A molecular weight distribution (*M_w_*/*M_n_*) of PBLA was determined to be 1.09 from the GPC chart (Figure S1B).

### 2.4 Synthesis of Polyaspartamide Derivative, PAsp­(DET/CHE)

PBLA (DP = 25, 20 mg) was dissolved in NMP (1 mL) and cooled to 10 °C. The PBLA solution was added dropwise to the mixture of DET (263 μL) and CHE (1.14 mL) (molar ratio = 1:3.2) in NMP (1 mL).^13,14,29^ The solution was stirred at 10 °C for 24 h under an argon atmosphere. The mixture was precipitated in diethyl ether. The precipitate was filtered and dissolved in ice-cooled 0.01 M HCl. The polymer was purified by stepwise dialysis against 0.01 M HCl and deionized water at 4 °C. The dialyzed solution was lyophilized to obtain the final product, PAsp­(DET/CHE). The conversion of benzyl ester groups to DET and CHE moieties in the side chains was confirmed in the ^1^H NMR spectrum from the peak intensity ratio between total protons in δ = 0.5–2.0 ppm and δ = 2.3–3.9 ppm.

### 2.5 Synthesis, Identification, and Purification of Circular RNAs

DNA encoding self-circularization P1 RNA construct for CVB3-FLuc with AC40 spacer (Table S1) was designed and synthesized by gBlocks service (Integrated DNA Technologies, Singapore) as described previously,^24^ and then inserted into the pTOP vector using an In-Fusion HD cloning kit (Takara Bio USA, Mountain View, CA, USA). The sequence of plasmid DNA was confirmed by the Sanger sequencing service (Cosmogenetech, Seoul, Republic of Korea). PCR DNA template for T7 *in vitro* transcription (IVT) was prepared by standard PCR using a thermal cycler and a Phusion Hot Start II High-Fidelity DNA polymerase (Thermo Fisher Scientific) using T7 G F and R primers (Table S1). PCR DNA templates were purified using a LaboPass gel extraction kit (Cosmogenetech).

IVT for self-circularization to generate circular RNAs was carried out using a HiScribe T7 High Yield RNA synthesis kit (New England Biolabs, Ipswich, MA, USA). The resulting IVT sample containing circular RNAs was purified using a Monarch RNA cleanup kit (New England Biolabs). RNase R (MCLAB, South San Francisco, CA, USA) treatment was performed according to the manufacturer’s protocol with minor modifications. Specifically, 120 μg of the IVT sample was incubated with 20 units of RNase R at 37 °C for 30 min. The RNase R-treated sample was purified again using a Monarch RNA cleanup kit.

RT-PCR was carried out to confirm precise circular RNA generation by checking sequence information on the splicing junction with STS F & R primers (Table S1) as described previously.^24^ The amplified specific band was extracted from agarose gel using a LaboPass gel extraction kit (Cosmogenetech), and then sequencing was performed by Cosmogenetech after TA cloning using a TOPcloner TA-Blunt kit (Enzynomics, Daejeon, Republic of Korea).

For *in vitro* and *in vivo* experiments, circular RNAs were purified by an ion-pair reversed-phase high-performance liquid chromatography (IP-RP HPLC) using a 1260 Infinity II bioinert LC system (Agilent Technologies, Santa Clara, CA, USA) and a 250 mm × 10 mm reverse-phase LC column with a particle size of 5 μm and a pore size of 4,000 Å (Agilent Technologies: PLRP-S 4,000 Å). RNA was purified with a linear gradient using buffer A (100 mM hexylammonium acetate [HAA] in 10% acetonitrile [ACN]; ADS Biotec, Omaha, NE, USA) and buffer B (82–95%, 100 mM HAA in 50% ACN; ADS Biotec) for 50 min at a flow rate of 1.8 mL/min. Column oven temperature was set at 60 °C. RNA was detected by UV absorption at 260 nm. Circular RNA fractions were recovered by a standard ethanol precipitation method. The recovered circular RNAs were dissolved in nuclease-free water, and then the concentration was determined using a NanoDrop microvolume UV–vis spectrophotometer (Thermo Fisher Scientific). Prepared RNAs were analyzed by denatured 4% polyacrylamide-7 M urea gel electrophoresis (PAGE) as described previously.^24^

### 2.6 Preparation and Characterization Linear or Circular RNA-Loaded Polyplexes

PAsp­(DET/CHE) were dissolved in HEPES buffer (10 mM, pH 7.3) and then mixed with FLuc linear or circular RNA solution (100 ng/μL RNAs in HEPES buffer) to prepare linear or circular RNA-loaded polyplexes (10 ng/μL RNAs) at the desired molar ratio of amino groups in the polymer to phosphate groups in RNA solution (*N*/*P* ratio). Polyplex sizes (cumulant diameter, *D*_H_), size distributions (polydispersity index (PDI)), and ζ potentials were determined by using a Zetasizer Pro (Malvern Instruments, Worcestershire, U.K.) equipped with a He–Ne laser (λ = 633 nm) and 173° detection angle. *D*_H_ and PDI of polyplex were measured in low volume quartz cuvette (ZEN2112, Malvern Instruments). The data from the rate of decay in the photon correlation function were analyzed using the cumulant method to obtain *D*_H_ and PDI. ζ Potential of polyplex was measured in a folded capillary cell (Malvern Instruments). The ζ potentials were calculated from the measured electrophoretic mobility according to the Smoluchowski equation.

### 2.7 Gel Retardation Assay

PAsp­(DET/CHE) and FLuc circular RNA were mixed at *N*/*P* ratios of 0, 1, 2, 3, 4, and 5, as aforementioned. Glycerol was added to the mixed solutions (final glycerol concentration: 0.08 vol %, final RNA amount: 200 ng). The samples were electrophoresed on an agarose gel (1 wt % agarose gel, 0.5× TAE buffer, 135 V). The circular RNA on the gel was stained with ethidium bromide (EtBr) and visualized using a gel imager (GelDoc, Biorad, CA, USA). To observe the stability of polyplex against PBS containing 10% FBS (PBS/FBS), polyplexes (*N*/*P* = 2.8, 100 ng, 10 μL) were mixed with PBS/FBS (90 μL) for 15 and 30 min at 37 °C. The mixture was purified with an RNeasy Mini Kit (Qiagen, Hilden, Germany). The samples were electrophoresed on an agarose gel (1 wt % agarose gel, 0.5× TAE buffer, 135 V). The RNA on the gel was stained with EtBr and visualized by using a gel imager. The band intensity was quantified using the ImageJ software.

### 2.8 Fluorescence Resonance Energy Transfer (FRET) for Polyplex Condensation

PAsp­(DET/CHE) was labeled with Alexa Fluor 546 NHS ester (Alexa546) (Thermo Fisher Scientific) in MeOH with a small amount of TEA to produce Alexa546-labeled polymers. Polyplexes were prepared by mixing Alexa546-labeled PAsp­(DET/CHE)­s and linear/circular Cy5-labeled RNAs (Cy5-RNAs) at *N*/*P* = 2.8 and incubating for 1 h. Fluorescence intensities of samples were measured using a spectrofluorometer (FP-8300, Jasco, Japan) with excitation at 505 nm and emission detected ranging from 550 to 750 nm. The FRET ratio of samples was calculated as the fluorescence intensity (FI) at 671 nm divided by FI at 575 nm.

### 2.9 NR Assay for Hydrophobicity Measurement

NR was dissolved to the concentration of 1 mM in DMSO and stored at −80 °C. PAsp­(DET/CHE) polymers were dissolved in HEPES buffer (10 mM, pH 7.3) at 190 μg/mL. The polymers were mixed with NR at a molar ratio of NR: polymer = 1:1.33. The mixture was incubated for 15 min, followed by mixing with linear/circular RNA (200 ng). The polyplexes were further incubated for 1 h. Fluorescence intensities of NR from samples were measured using a spectrofluorometer (FP-8300) with excitation at 530 nm and detected emission ranging from 550 to 750 nm.

### 2.10 Luciferase Linear/Circular RNA Transfection in Cultured Immune Cells

Jurkat, NK-92 mi, or RAW 264.7 cells were seeded into a 96-well plate at a density of 8,000 cells/well in culture medium, respectively. Polyplex solutions prepared from linear or circular RNA were added to each well (100 ng of RNA/well, *N*/*P* = 2.8) and incubated for desired amounts of time. Jurkat and NK-92 mi cells in each well were transferred to microcentrifuge tubes and washed with PBS twice. The cells were lysed using passive lysis buffer (Promega, Madison, WI, USA) in the tube. RAW 264.7 cells were washed with PBS twice and similarly lysed by using a passive lysis buffer in the plate. The photoluminescence intensity of the cell lysate was measured using a Luciferase Assay System (Promega) in a multimode microplate reader (Spark, Tecan Trading AG, Switzerland).

### 2.11 Flow Cytometric Assay

Jurkat, NK-92 mi, or RAW 264.7 cells were seeded into a 24-well plate at a density of 50,000 cells/well in culture medium, respectively. Polyplex solutions containing linear/circular Cy5-RNAs were added to each well (500 ng of RNA/well, *N*/*P* = 2.8) and incubated for 4 h. The fluorescence intensity of Cy5-RNA derived from the cells was measured using flow cytometry (Attune CytPix, Thermo Fisher Scientific), where the cells were excited with a 633 nm laser.

### 2.12 Animal Experiments

All animal experiments were conducted under the Guidelines for the Care and Use of Laboratory Animals of HLB bioStep Co., Ltd. (Incheon, Republic of Korea). Balb/c mice (female, 6-week old, Koatech, Gyeonggi-do, Republic of Korea) were intravenously injected with a single dose of PAsp­(DET/CHE) polyplex (3 μg RNA/shot, *N*/*P* = 2.8) containing FLuc linear/circular RNAs or saline. At 4, 12, 24, and 48 h postinjection, the mice were anesthetized with isoflurane and intraperitoneally injected with Luciferin (150 mg/kg, 200 μL). The mice were measured by the IVIS Spectrum (Revvity, MA, USA) with a 1 min exposure time, 8 binning, and 1 F/stop. After being measured by the IVIS Spectrum at 48 h postinjection, the mice were sacrificed and perfused with PBS. Various organs were incubated in Luciferin solution for 10 min, and the photoluminescence intensities of the organs were measured by the IVIS Spectrum.

For polyplex accumulation in splenic immune cells, C57BL/6 mice (female, 6-week old, Koatech, Gyeonggi-do, Republic of Korea) were intravenously injected with a single dose of PAsp­(DET/CHE) polyplex (3 μg Cy5-RNA/shot, *N*/*P* = 2.8) containing linear/circular Cy5-RNA or saline. The mice were sacrificed at 4 h postinjection. The spleen was collected and stored in RPMI 1640. The spleen was transferred into spleen dissociation buffer (Spleen dissociation kit, 130-095-926, Miltenyi Biotec, Germany) and dissociated into single cells using a dissociator (gentleMACS, Miltenyi Biotec). Debris was removed using a strainer, and the cell suspension was treated with red blood cell (RBC) lysis buffer at room temperature for 15 min to remove RBCs. The cells were dispersed in RPMI 1640 containing 10% FBS. The splenic cells were prepared at a concentration of 1 × 10^6^ cells in FACS staining buffer (50 μL) and stained with surface marker antibodies for 30 min at 4 °C in the dark (Tables S2 and S3). The cells were then washed twice with FACS staining buffer and resuspended in PBS. The cells were analyzed using a NovoCyte 3000 (Agilent, CA, USA).

### 2.13 Statistical Analysis

Statistical analyses of data in Figures 4 and 5 were performed using parametric multiple comparison procedures, including One-way ANOVA and Dunnett’s multiple comparison test in Prism 10.3.1 (GraphPad Software Inc., CA, USA).

## 3 Results and Discussion

### 3.1 Synthesis of PAsp­(DET/CHE) and Circular RNA

An amphiphilic polyaspartamide derivative, PAsp­(DET/CHE), was synthesized by the aminolysis reaction of a parent polymer, PLBA. PBLA was synthesized through ring-opening polymerization using a monomer BLA-NCA with an initiator, *n*-butylamine.^27,28^ The polymerization was performed at 37 °C for 72 h under an argon atmosphere. The polymer was purified using hexane/ethyl acetate precipitation to obtain a white powder. The resulting PBLA was characterized by using ^1^H NMR and GPC analyses. The degree of polymerization (DP) of the BLA units in PBLA was 25 (Figure S1A), and the molecular weight distribution (*M_w_*/*M_n_*) was 1.09 (Figure S1B). Then, the mixture of DET and CHE (molar ratio = 1:3.2) was reacted with PBLA at 10 °C for 24 h under an argon atmosphere.^13,14,29^ The mixture was precipitated in diethyl ether and further purified by using dialysis to obtain PAsp­(DET/CHE). ^1^H NMR spectroscopy of the polymer revealed that the amounts of DET and CHE moieties were 10 and 15, respectively (Figure S2). The DET moiety exhibits acidic pH-selective membrane disruption through a distinctive change in protonation status from the membrane-inert monoprotonated state at physiologically neutral pH (∼7.4) to the membrane-active deprotonated state at acidic endosomal pH (∼5.5).^27,28^ The CHE moiety provides enhanced particle stability in physiological buffers, such as blood and cell culture media, via hydrophobic interactions during polyplex formation, selected from various structures of alkyl groups.^13,14^

Circular RNAs containing CVB3-FLuc as a gene of interest (GOI) were prepared using the end-to-end STS method and the P1 RNA construct, as described previously^24^ (Figure 1A). GOI sequences downstream of the target site were inserted adjacent to the group I intron ribozyme to design the P1 RNA construct for self-circularization. After self-circularization during *in vitro* transcription, GOI sequences can reconnect with other parts of the GOI, resulting in a complete GOI in the generated circular RNAs (Figure 1A). Unlike FLuc linear RNAs (or FLuc mRNAs) with 5 moU nucleotides, CVB3-FLuc circular RNAs (FLuc circular RNAs) were prepared by using only natural nucleotides. The generated FLuc circular RNAs were analyzed using 4% denatured PAGE with or without RNase R treatment, which enriched circular RNAs by degrading linear byproducts (Figure 1B). The generated circular RNAs were purified using IP-RP HPLC for *in vitro* and *in vivo* experiments (Figures S3 and 1B). Circular RNAs generated by precise self-circularization through the end-to-end STS reaction were confirmed by the sequencing of specific splicing junctions using RT-PCR products (Figure 1C). Purified circular RNAs were used in subsequent experiments.

**Figure 1:**
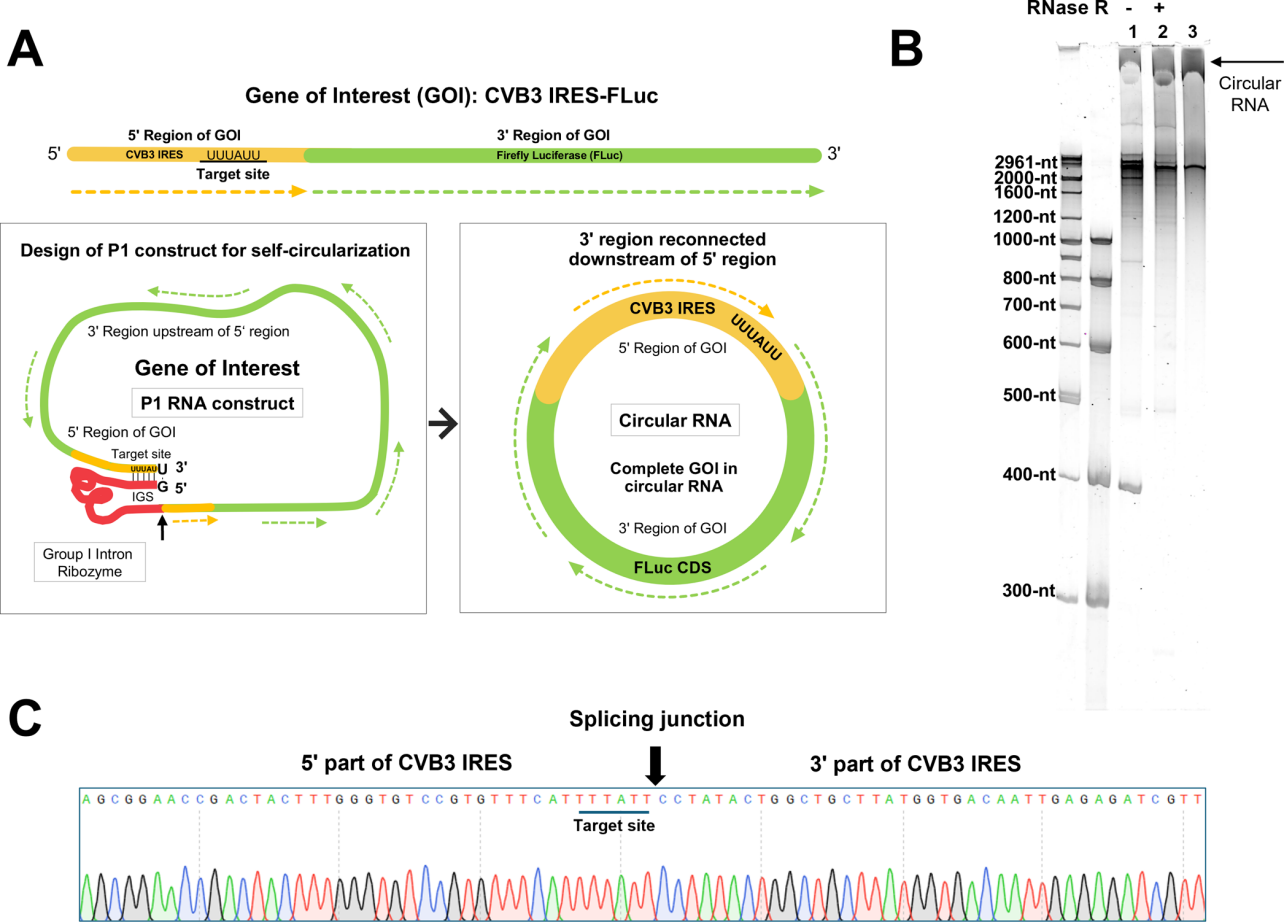
(A) Design of P1 RNA construct for self-circularization to generate circular RNA (CVB3-FLuc) using end-to-end STS reaction. Split GOI parts can be reconnected in the correct order after self-circularization. The black arrow indicates the cleavage site for transesterification. (B) Denatured PAGE (4%) analysis of *in vitro*-transcribed RNAs with or without RNase R treatment (Lane 1 and 2, respectively). HPLC-purified circular RNAs in lane 3. (C) Sequencing analysis of RT-PCR product of circular RNAs prepared using the P1 construct. The specific splicing junction is shown by an arrow.

### 3.2 Preparation and Characterization of Linear/Circular RNA-Loaded Polyplexes

Polyplexes were formed by mixing PAsp­(DET/CHE) with linear or circular RNAs at the desired *N*/*P* ratios. Polyplex samples were prepared using FLuc circular RNAs at increasing *N*/*P* ratios in a HEPES buffer (10 mM, pH 7.3). Agarose gel electrophoresis was used to visualize circular RNA encapsulation in the polyplexes (Figure S4). The disappearance of the free circular RNA band at *N*/*P* ≥ 2 in all polyplexes indicated that the circular RNA was fully complexed with the polymers at the *N*/*P* ratios, similarly to linear RNA in the previous studies.^13,14^ Considering the approximately 50% protonation degree of the amino groups in the DET moieties at pH 7.3, an *N*/*P* ratio of 2 corresponds to the charge-neutralization point between the protonated amines in the polymers and the phosphates in the linear/circular RNA.^27,28^ Thus, the gel electrophoresis results demonstrated the successful formation of polyplexes between PAsp­(DET/CHE) and FLuc circular RNAs.

Hydrodynamic diameter (*D*_H_), polydispersity index (PDI), and ζ potential of polyplexes at *N*/*P* = 2.8 were determined using a Zetasizer (Table 1). PAsp­(DET/CHE) polyplexes containing linear mRNA induced *N*/*P*-dependent mRNA expression shift from the spleen to the lung, ranging from *N*/*P* = 2–5, with the polyplex at *N*/*P* = 2.8 exhibiting the highest linear mRNA expression in the spleen.^16^ The *D*_H_ values of the FLuc linear RNA (5 moU nucleotide)-loaded polyplexes were approximately 117 nm, whereas those of the FLuc circular RNA (natural nucleotide)-loaded polyplexes exhibited similar *D*_H_ values of approximately 113 nm with a relatively narrow PDI of approximately 0.28 (Figure 2A). Considering that the lengths of the linear and circular RNAs were similar (1,922 and 2,434 nucleotides, respectively), the circular structure of the RNAs did not significantly affect the particle sizes of the PAsp­(DET/CHE) polyplexes. Similar ζ potentials (29 ± 3 and 31 ± 3 mV) were obtained for linear and circular RNA polyplexes, respectively, regardless of their RNA lengths and structure. Overall, these results indicated the successful formation of circular RNA-loaded polyplexes of PAsp­(DET/CHE) in HEPES buffer (pH 7.3), similar to that of the linear RNA-loaded polyplexes.

**Figure 2:**
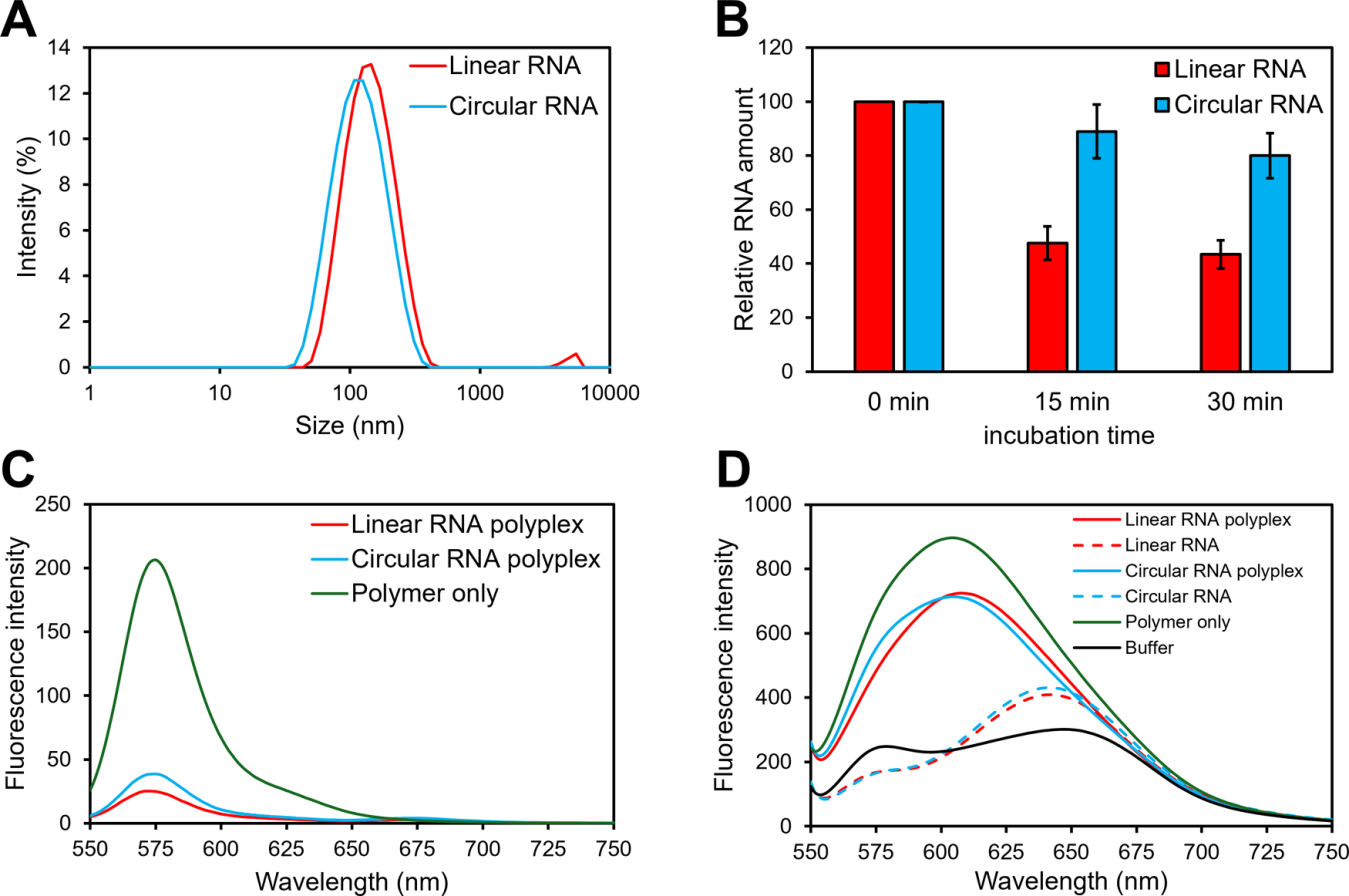
(A) DLS histograms of PAsp­(DET/CHE) polyplexes (*N*/*P* = 2.8) prepared with FLuc linear (5 moU nucleotides) and circular (natural nucleotides) RNAs. (B) Quantitative RNA amounts after incubation of polyplex (*N*/*P* = 2.8) in 10% FBS at 37 °C. After incubation, the polyplex samples were purified with an RNeasy mini kit for RNA release (*n* = 3). (C) Fluorescence emission profile of Alexa546-labeled PAsp­(DET/CHE) polymer and its polyplex containing linear/circular Cy5-RNA. (D) NR fluorescence emission profiles of linear and circular RNA polyplexes (*N*/*P* = 2.8).

**Table 1:** D_H_, PDI, ζ Potential of FLuc Linear and Circular RNA-Loaded Polyplexes (Mean ± SD, *n* = 3)

polyplex (*N*/*P* = 2.8)	*D*_H_ (nm)	PDI	ζ potential (mV)
linear RNA polyplex	117 ± 2	0.24 ± 0.01	29 ± 3
circular RNA polyplex	113 ± 11	0.28 ± 0.01	31 ± 3

We then evaluated the stability of the polyplexes in the presence of FBS to understand the polyplex formation of linear and circular RNAs. Polyplex samples (*N*/*P* = 2.8) were incubated with PBS containing 10% FBS at 37 °C for 15 and 30 min. Next, the mixture was purified to obtain intact linear or circular RNAs, which were then visualized by using gel electrophoresis (Figure S5). Higher amounts of intact circular RNA than linear RNA were observed after incubation for 15 min (Figure 2B). Considering that complex formation between RNAs and PAsp­(DET/CHE) was not tight at *N*/*P* = 2.8, the circular RNA itself, rather than polyplex stability, might be more resistant to FBS conditions than the linear RNA.^30^

We compared the FRET intensities of the polyplex (*N*/*P* = 2.8) using linear and circular Cy5-RNAs and Alexa546-labeled PAsp­(DET/CHE) to elucidate RNA condensation states (Figures 2C and S6). Both linear and circular RNA-loaded polyplexes exhibited high FRET intensities relative to the background signals. The FRET ratios (defined as the fluorescence intensity (FI) at 671 nm/FI at 575 nm) of the linear and circular RNA-loaded polyplexes were 0.104 and 0.103, respectively. This indicated that the RNA condensation states of both polyplexes were similar, regardless of the RNA structure. We further investigated hydrophobic interactions within the polyplexes using NR. To this end, PAsp­(DET/CHE) was dissolved in HEPES buffer (10 mM, pH 7.3) and incubated with the NR for 15 min. Then, the mixture was mixed with FLuc linear/circular RNAs and further incubated for an additional 1 h. The NR fluorescence intensities of the polyplexes were measured using a spectrofluorometer with excitation at 530 nm and emission at 550–750 nm. Notably, the NR FI of the PAsp­(DET/CHE) polymer was higher than that of the linear and circular RNA-loaded polyplexes (Figure 2D). Amphiphilic PAsp­(DET/CHE)­s in the absence of RNAs were inter- and intramolecularly associated via hydrophobic interactions of the CHE moieties, inducing high NR fluorescence. The hydrophobic association may be released during the electrostatic interaction between RNAs and PAsp­(DET/CHE)­s during polyplex formation, which decreased the NR fluorescence. This is sharply contrasted with polyplex formation at high *N*/*P* ratios (e.g., *N*/*P* = 5) in a previous study, where NR fluorescence increased owing to enhanced hydrophobic interactions.^31^

### 3.3 Evaluation of *In Vitro* Circular RNA Transfection Efficiency of Polyplexes

The efficiency of FLuc linear (5 moU nucleotides) and circular (natural nucleotides) RNA delivery by the polyplexes was determined in cultured human T cells, natural killer (NK) cells, and macrophages, respectively. PAsp­(DET/CHE) was mixed with FLuc linear or circular RNAs at *N*/*P* = 2.8 to form polyplex samples. The polyplex samples were then added to Jurkat, NK-92 mi, or RAW 264.7 cells at 100 ng of RNA/well, and the cells were incubated for the desired period of time. Following luciferin treatment, time-dependent FLuc expression was measured using a luminescence plate reader (Figure 3A–C). Linear RNA-loaded polyplexes exhibited luminescence intensities higher than those of circular RNA-loaded polyplexes at all incubation times in Jurkat and RAW 264.7 cells. However, both polyplexes showed marginal luciferase expression in NK-92 mi cells, which was 10^3^–10^5^-fold lower than that in Jurkat and RAW 264.7 cells. Next, we assessed the cellular uptake of the polyplexes using flow cytometry with Cy5-RNAs to investigate the mechanism underlying the significantly higher FLuc linear RNA expression in immune cells. Jurkat, NK-92 mi, and RAW 264.7 cells were incubated with linear or circular naked Cy5-RNA or Cy5-RNA-loaded polyplexes (*N*/*P* = 2.8) for 4 h prior to flow cytometric analysis (Figures 3D and S7). The Cy5 fluorescence intensities were similar between the linear and circular Cy5-RNAs (Figure S8), indicating that higher FLuc expression in Jurkat and RAW 264.7 cells treated with linear RNA-loaded polyplexes might be due to more efficient binding of ribosomes in the capping structure of linear RNA than in the CVB3 IRES of circular RNA. In addition, the translation efficiency of IRES is significantly affected by the cell type.^32^ Notably, the cellular uptake of linear and circular RNA-loaded polyplexes in NK-92 mi cells was significantly lower than that in Jurkat and RAW 264.7 cells. This indicated that the marginal FLuc expression levels in NK-92 mi cells were mainly due to the low cellular uptake of polyplexes.^33^

**Figure 3:**
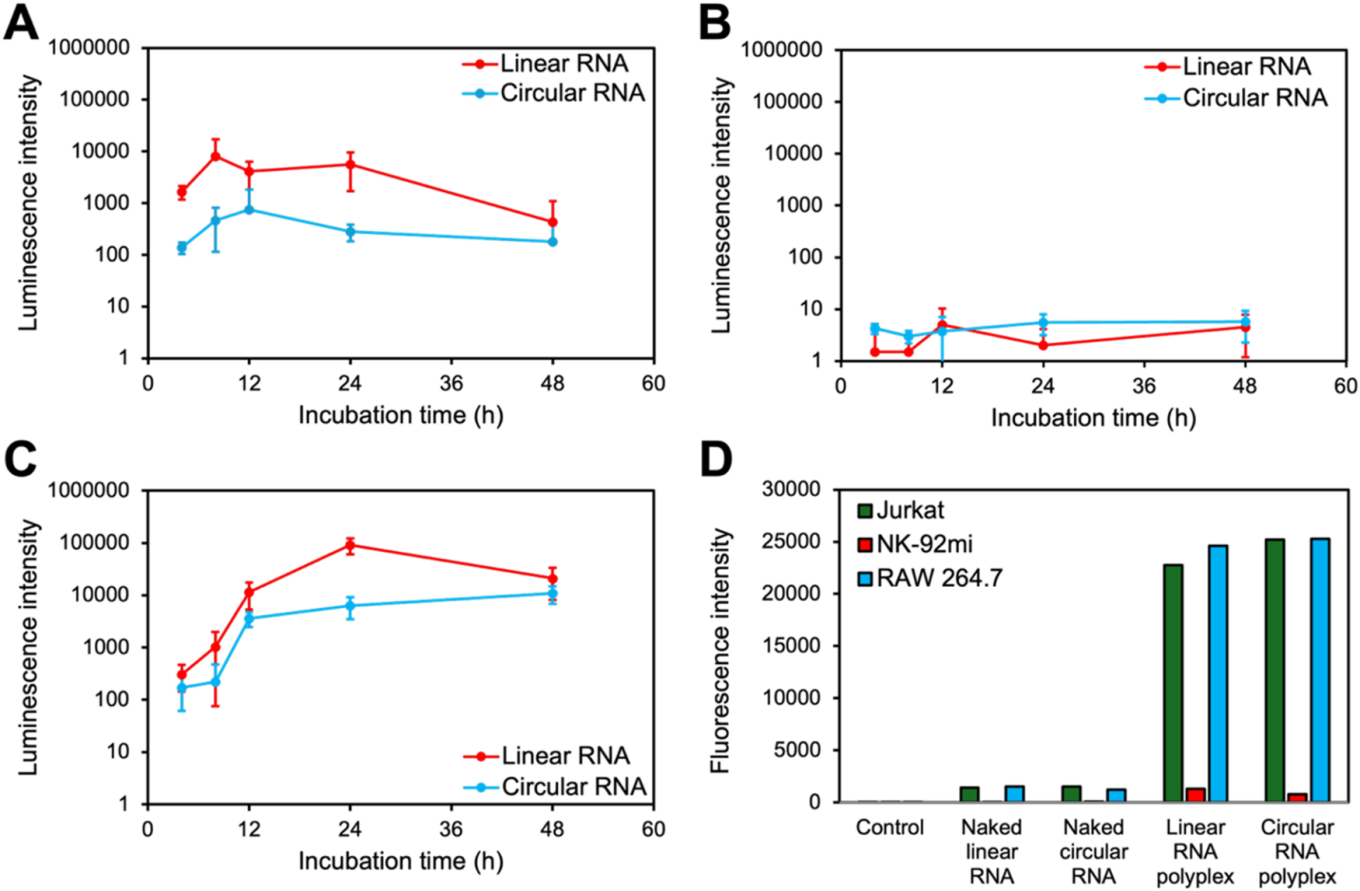
Luminescence intensities of (A) Jurkat, (B) NK-92 mi, and (C) RAW 264.7 cells transfected with FLuc linear or circular RNA-loaded PAsp­(DET/CHE) polyplexes (*N*/*P* = 2.8, 100 ng RNA/well) for 24 h. Results are expressed as mean ± SD (*n* = 4). (D) Cellular uptake of naked Cy5-RNA and Cy5-RNA-loaded PAsp­(DET/CHE) polyplexes (*N*/*P* = 2.8, 500 ng Cy5-RNA/well) into Jurkat, NK-92 mi, and RAW 264.7 cells after incubation for 4 h, measured using flow cytometry.

### 3.4 Biodistribution and Splenic Immune Cell Accumulation of Polyplexes

We compared the delivery efficiency of the linear and circular RNA-loaded PAsp­(DET/CHE) polyplexes into the spleen after intravenous administration. FLuc linear (5 moU nucleotides) or circular (natural nucleotides) RNA-loaded polyplexes (3 μg RNA/mouse, *N*/*P* = 2.8, one injection) or saline were intravenously injected into BALB/c mice. The luminescence intensities of the mice were measured using an IVIS Spectrum at designated times. Both polyplexes primarily induced luciferase expression in the spleen (Figure 4A,B). The splenic luminescence intensities gradually decreased over time but remained higher than those of PBS-treated controls at 48 h. Notably, polyplexes loaded with linear RNA induced higher luciferase expression than those loaded with circular RNA at 4 and 12 h. However, luciferase expression levels were comparable at 24 h and were higher in the circular RNA group after 48 h, suggesting the inherently sustained protein expression capability of circular RNA. The mice were sacrificed 48 h postinjection, and the luminescence intensities of the excised organs were measured *ex vivo* using the IVIS Spectrum (Figures 4C and S9). Similarly, most FLuc linear and circular RNAs were expressed in the spleen.

**Figure 4:**
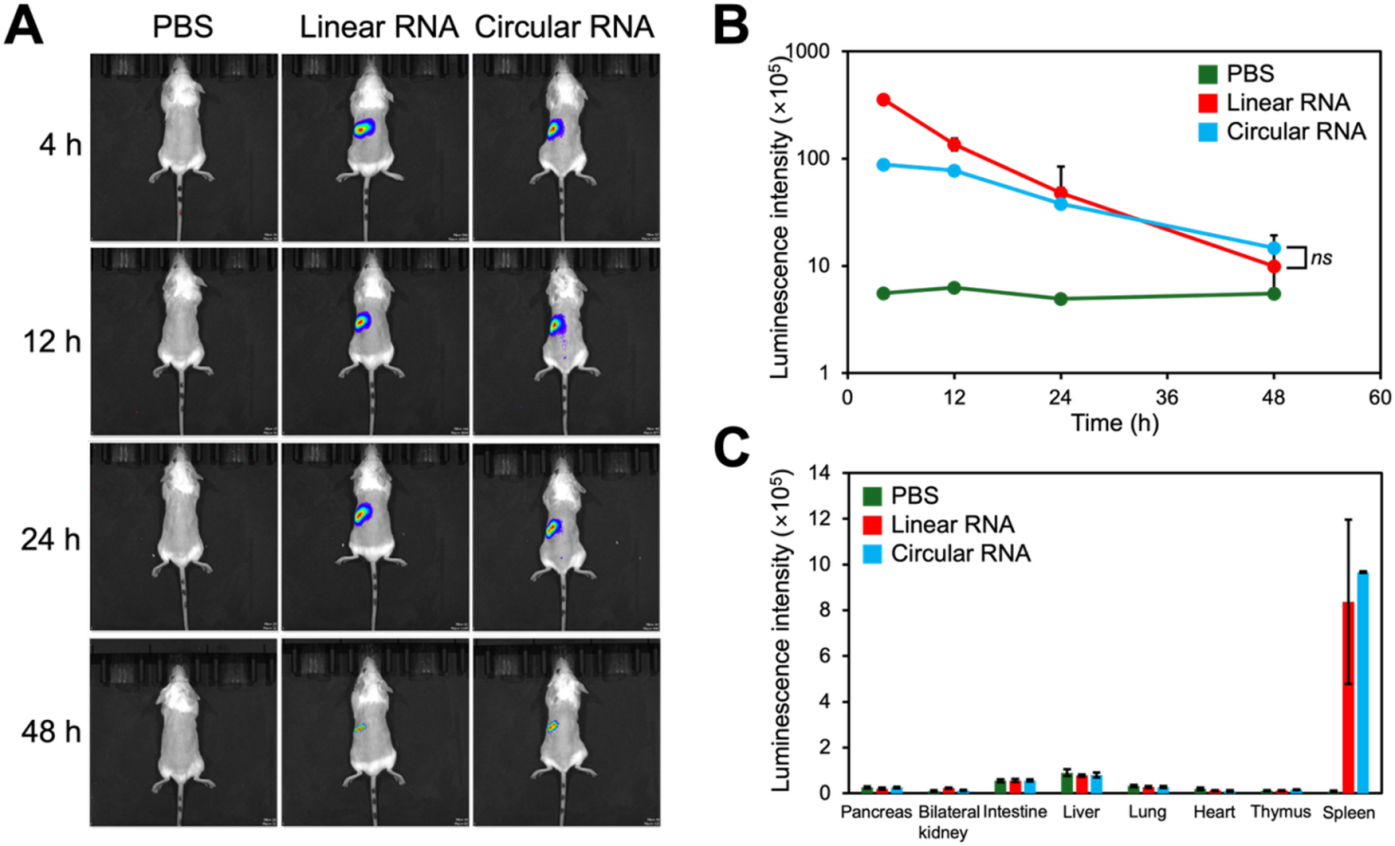
(A) Representative IVIS images and (B) time-dependent luminescence intensities of mice injected with PBS and linear/circular RNA-loaded polyplexes (3 μg RNA/shot, *N*/*P* = 2.8, one injection). (C) Luminescence intensities of various organs excised from the mice 48 h after intravenous administration. Results are expressed as mean ± SD (*n* = 4 for PBS-treated controls and *n* = 8 for linear/circular RNA-loaded polyplex-treated groups). The “ns” indicates statistically no significant difference.

We further investigated the accumulation of Cy5-RNA in splenic immune cells to examine the difference in the delivery efficacy between the linear and circular RNA-loaded polyplexes. FLuc linear/circular Cy5-RNA-loaded polyplexes (3 μg RNA/mouse, *N*/*P* = 2.8, one injection) or saline were intravenously injected into C57BL/6 mice. The Cy5 fluorescence intensities from various splenic immune and endothelial cells were analyzed 4 h postinjection using flow cytometry after staining with surface marker antibodies (Figure 5). All cell types in the spleen treated with both polyplexes exhibited significantly higher Cy5 fluorescence intensities than those treated with PBS, indicating the efficient delivery efficacy of the PAsp­(DET/CHE) polyplex in the spleen regardless of the payload. Notably, circular RNA-loaded polyplex accumulation was ∼3-fold higher in CD4+ T and CD8+ T cells than in linear RNA-loaded polyplexes but lower in dendritic cells and macrophages. Both polyplexes showed similar accumulation in the NK and B cells. Considering that dendritic cells and macrophages are mononuclear phagocytes, both cells more efficiently engulf micrometer-sized nanoparticles. Circular RNA-loaded polyplexes may maintain their innate nanometer sizes after intravenous administration due to their rigid circular RNAs, avoiding uptake into dendritic cells and macrophages. Considering that 14% of the total splenic cells were CD4+/CD8+ T cells (Table S4), the results indicated that the circular RNA-loaded PAsp­(DET/CHE) polyplexes have a relatively higher potential for *in vivo* CAR T therapy. Recent research achieved high mRNA expression in CD8+ T cells using anti-CD8 antibody-conjugated LNPs, accumulating ∼35 and ∼15% of CD8+ T cells in the spleen and lymph nodes, respectively.^12^

**Figure 5:**
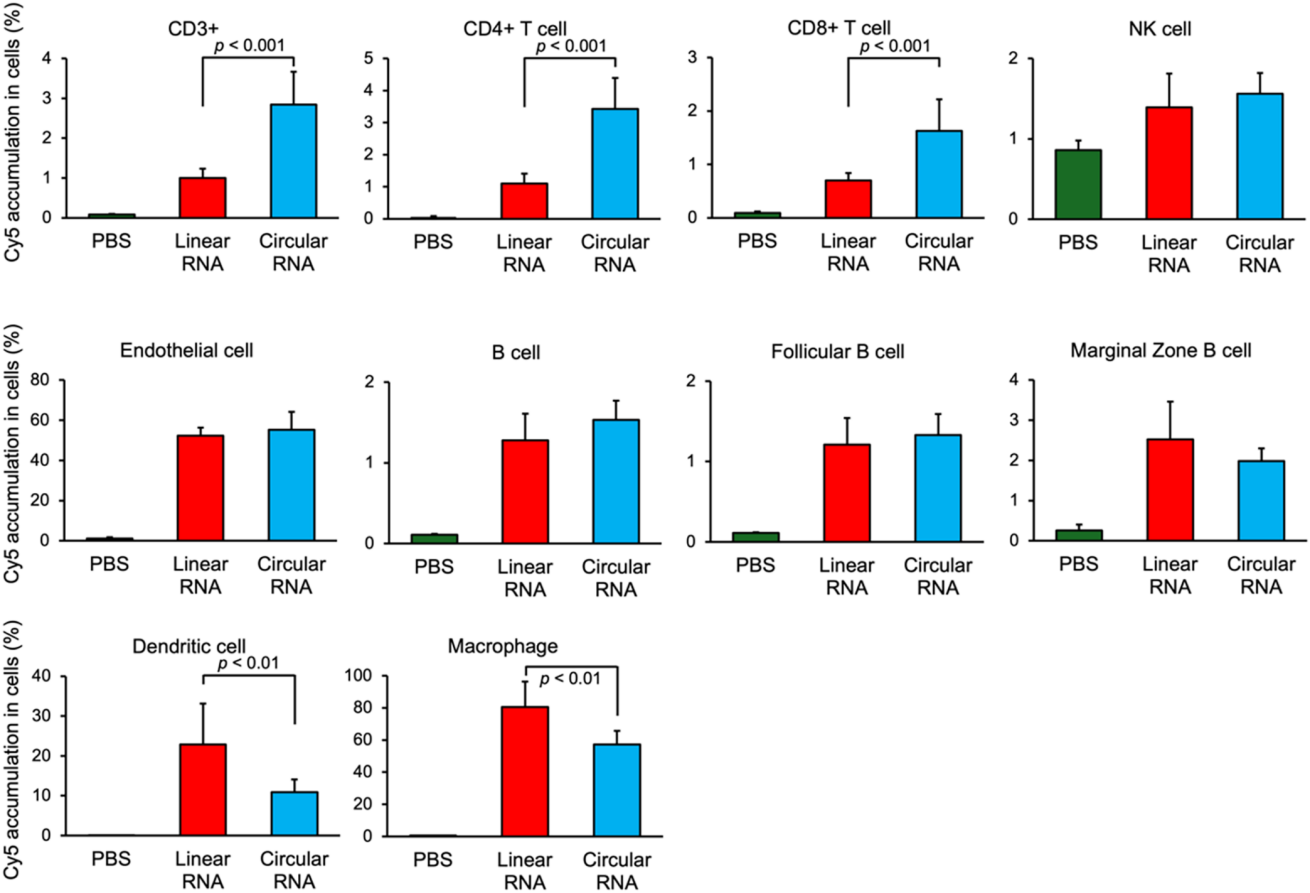
Accumulation of Cy5-RNAs in the splenic immune cells 4 h after intravenous administration of PBS and linear/circular Cy5-RNA-loaded polyplexes (3 μg RNA/shot, *N*/*P* = 2.8, one injection). Each % represents the proportion of Cy5-accumulated cells among each specific type of cell. Results are expressed as mean ± SD (*n* = 4 for PBS-treated controls and *n* = 8 for linear/circular RNA-loaded polyplex-treated groups).

## 4 Conclusions

In this study, the amphiphilic cationic polyaspartamide derivative, PAsp­(DET/CHE), was used for the polyplex-mediated delivery of FLuc linear and circular RNAs into the spleen via intravenous administration. Both polyplexes at *N*/*P* = 2.8 exhibited similar physicochemical characteristics, such as a *D*_H_ value of 110–120 nm, a ζ potential of ∼30 mV, and a relatively low PDI of <0.3, regardless of the RNA payload. Notably, the inter/intramolecular hydrophobic interactions in the PAps­(DET/CHE) polymers decreased during polyplex formation, indicating that the electrostatic interaction between the polymer and linear/circular RNA was the main driving force for polyplex formation at *N*/*P* = 2.8. FLuc linear RNA-loaded polyplexes more efficiently induced FLuc expression than circular RNA-loaded polyplexes in Jurkat and RAW 264.7 cells, probably due to higher ribosome binding to the capping structure of mRNA or cell type dependency of IRES.^32^ PAsp­(DET/CHE) polyplexes efficiently delivered FLuc linear/circular RNAs into the spleen after intravenous administration and induced high luciferase expression for up to 48 h. Notably, circular RNA (natural nucleotides)-loaded polyplexes showed longer-lasting FLuc expression patterns than linear RNA (5 moU nucleotides)-loaded polyplexes at 48 h *in vivo*, unlike *in vitro* cell-based experiments. In immune cells, the current circular RNA construct with CVB3 IRES exhibited weaker translation efficiency than linear RNA, despite showing better expression stability. Viral IRESs are classified into distinct groups (I–IV) that require different initiation factors or IRES-trans-acting factors, making IRES-driven translation highly dependent on cell type-specific factor expression.^34^ Therefore, to increase the translation efficiency of circular RNA in immune cells, IRES screening that drives robust translation in immune cells is required for therapeutic CAR applications. In addition, coding DNA sequence optimization will be pursued to minimize structural interference with the IRES, thereby improving the translation efficiency. Ultimately, FLuc circular Cy5-RNA-loaded polyplexes demonstrated a higher T cell delivery efficacy with relatively lower accumulation in dendritic cells and macrophages than linear Cy5-RNA-loaded polyplexes. Therefore, this optimized polyplex formulation with circular RNA may be utilized as an *in vivo* CAR RNA delivery platform for future T cell therapies.

## Supplementary Material



## Notes

Additional experimental details, including DNA sequence, protein marker, and fluorescence for immune cell staining; ^1^H NMR spectra and GPCs of synthetic materials and circular RNAs, agarose gel electrophoresis, cellular uptake profiles, fluorospectrometric and flow cytometric analysis, and IVIS images of mouse organs (PDF)
