# Supplementary material for: Delivery of Circular RNAs into Splenic Immune Cells via Intravenous Administration of Polyaspartamide Derivative Polyplexes
Source: ACS Biomater Sci Eng. 2026 Mar 27;12(4):2243–53. doi: 10.1021/acsbiomaterials.5c02147 (PMC13080761; doi:10.1021/acsbiomaterials.5c02147)
Supplement: Supplementary file 1 [file ab5c02147_si_001.pdf]

Supporting Information

Delivery of Circular RNAs into Splenic Immune Cells via Intravenous Administration of Polyaspartamide Derivative Polyplexes

Jun Su An[1,a], Sung Been Lim[1,a], Kyung Hyun Lee[2,a], Seongcheol Kim[2], SeonJeong Kim[1], Jieun Lee[1], Dongsu Kim[2], Seung Ryul Han[2], Mitsuru Naito[3,4], Kanjiro Miyata[5], Hyun Jin Kim[1,6,7,8,*], Seong-Wook Lee[2,9,*]

1. Department of Biological Sciences and Bioengineering, Inha University, 100 Inha-ro, Michuhol-gu, Incheon, 22212, Republic of Korea
2. R&D Center, Rznomics Inc., Seongnam 13486, Republic of Korea
3. Department of Materials Science and Technology, Faculty of Advanced Engineering, Tokyo University of Science, 6-3-1 Niijuku, Katsushika-ku, Tokyo 125-8585, Japan
4. Center for Advanced Modalities and DDS, The University of Osaka, 1-10 Yamadaoka, Suita-shi, Osaka 565-0871, Japan
5. Department of Materials Engineering, Graduate School of Engineering, The University of Tokyo, 7-3-1 Hongo, Bunkyo-ku, Tokyo 113-8656, Japan
6. Department of Medicinal Biosciences and Bioengineering, Inha University, 100 Inha-ro, Michuhol-gu, Incheon 22212, Republic of Korea
7. Department of Biological Engineering, College of Engineering, Inha University, 100 Inha-ro, Michuhol-gu, Incheon 22212, Republic of Korea
8. Biohybrid Systems Research Center, Inha University, 100 Inha-ro, Michuhol-gu, Incheon 22212, Republic of Korea
9. Department of Bioconvergence Engineering, Research Institute of Advanced Omics, Dankook University, Yongin 16890, Republic of Korea

[a]These authors are equally contributed to this work.

*Corresponding authors:

Hyun Jin Kim: kimhyunjin@inha.ac.kr

Seong-Wook Lee: swl0208@dankook.ac.kr or swl0208@rznomics.com

Table S1. DNA sequences used in this study

DNA	Sequence (5' to 3')
CVB3-FLuc with AC40 spacer	TTAAAACAGCCTGTGGGTTGATCCCACCCACAGGCCCATTGGGCGCTAGCACTCTG<br>GTATCACGGTACCTTTGTGCGCCTGTTTTATACCCCCTCCCCCAACTGTAACTTAGA<br>AGTAACACACACCGATCAACAGTCAGCGTGGCACACCAGCCACGTTTTGATCAAG<br>CACTTCTGTTACCCCGGACTGAGTATCAATAGACTGCTCACGCGGTTGAAGGAGAA<br>AGCGTTCGTTATCCGGCCAACTACTTCGAAAAACCTAGTAACACCGTGGAAGTTGC<br>AGAGTGTTTCGCTCAGCACTACCCCAGTGTAGATCAGGTCGATGAGTCACCGCATT<br>CCCCACGGGCGACCGTGGCGGTGGCTGCGTTGGCGGCCTGCCCATGGGGAAACCC<br>ATGGGACGCTCTAATACAGACATGGTGCGAAGAGTCTATTGAGCTAGTTGGTAGTC<br>CTCCGGCCCCTGAATGCGGCTAATCCTAACTGCGGAGCACACACCCTCAAGCCAG<br>AGGGCAGTGTGTCGTAACGGGCAACTCTGCAGCGGAACCGACTACTTTGGGTGTC<br>CGTGTTTCATTTTATTCCTATACTGGCTGCTTATGGTGACAATTGAGAGATCGTTACC<br>ATATAGCTATTGGATTGGCCATCCGGTGACTAATAGAGCTATTATATATCCCTTTGTTG<br>GGTTTATACCACTTAGCTTGAAAGAGGTTAAAACATTACAATTCATTGTTAAGTTGA<br>ATACAGCAAAATGGAGGACGCCAAGAACATCAAGAAGGGCCCCGCCCCTTCTAC<br>CCCCTGGAGGACGGCACCGCCGGCGAGCAGCTGCACAAGGCCATGAAGCGGTAC<br>GCCCTGGTGCCCGGCACCATCGCCTTCACCGACGCCCACATCGAGGTGGACATCAC<br>CTACGCCGAGTACTTCGAGATGAGCGTGCGGCTGGCCGAGGCCATGAAGCGGTAC<br>GGCCTGAACACCAACCACCGGATCGTGGTGTGCAGCGAGAACAGCCTGCAGTTCT<br>TCATGCCCGTGCTGGGCGCCCTGTTCATCGGCGTGGCCGTGGCCCCCGCCAACGAC<br>ATCTACAACGAGCGGGAGCTGCTGAACAGCATGGGCATCAGCCAGCCCACCGTGG<br>TGTTCGTGAGCAAGAAGGGCCTGCAGAAGATCCTGAACGTGCAGAAGAAGCTGC<br>CCATCATCCAGAAGATCATCATCATGGACAGCAAGACCGACTACCAGGGCTTCCAG<br>AGCATGTACACCTTCGTGACCAGCCACCTGCCCCCCGGCTTCAACGAGTACGACTT<br>CGTGCCCGAGAGCTTCGACCGGGACAAGACCATCGCCCTGATCATGAACAGCAGC<br>GGCAGCACCGGCCTGCCCAAGGGCGTGGCCCTGCCCCACCGGACCGCCTGCGTGC<br>GGTTCAGCCACGCCCGGGACCCCATCTTCGGCAACCAGATCATCCCCGACACCGCC<br>ATCCTGAGCGTGGTGCCCTTCCACCACGGCTTCGGCATGTTCACCACCCTGGGCTA<br>CCTGATCTGCGGCTTCCGGGTGGTGCTGATGTACCGGTTCGAGGAGGAGCTGTTCC<br>TGCGGAGCCTGCAGGACTACAAGATCCAGAGCGCCCTGCTGGTGCCCACCCTGTT<br>CAGCTTCTTCGCCAAGAGCACCCTGATCGACAAGTACGACCTGAGCAACCTGCAC<br>GAGATCGCCAGCGGCGGCGCCCCCCTGAGCAAGGAGGTGGGCGAGGCCGTGGCC<br>AAGCGGTTCCACCTGCCCGGCATCCGGCAGGGCTACGGCCTGACCGAGACCACCA<br>GCGCCATCCTGATCACCCCCGAGGGCGACGACAAGCCCGGCGCCGTGGGCAAGGT<br>GGTGCCCTTCTTCGAGGCCAAGGTGGTGGACCTGGACACCGGCAAGACCCTGGGC<br>GTGAACCAGCGGGGCGAGCTGTGCGTGCGGGGCCCCATGATCATGAGCGGCTACG<br>TGAACAACCCCGAGGCCACCAACGCCCTGATCGACAAGGACGGCTGGCTGCACA<br>GCGGCGACATCGCCTACTGGGACGAGGACGAGCACTTCTTCATCGTGGACCGGCT<br>GAAGAGCCTGATCAAGTACAAGGGCTACCAGGTGGCCCCCGCCGAGCTGGAGAGC<br>ATCCTGCTGCAGCACCCCAACATCTTCGACGCCGGCGTGGCCGGCCTGCCCGACG<br>ACGACGCCGGCGAGCTGCCCGCCGCCGTGGTGGTGCTGGAGCACGGCAAGACCAT<br>GACCGAGAAGGAGATCGTGGACTACGTGGCCAGCCAGGTGACCACCGCCAAGAA<br>GCTGCGGGGCGGCGTGGTGTTCGTGGACGAGGTGCCCAAGGGCCTGACCGGCAA<br>GCTGGACGCCCGGAAGATCCGGGAGATCCTGATCAAGGCCAAGAAGGGCGGCAA<br>GATCGCCGTGTGAAAAAAACAAAAAACAAAACAAAAACAAAAAACAAAAAAAA
T7 G F primer	ATAATACGACTCACTATAGGGG
R primer	AATAAAATGAAACACGGACACCCAA
STS F primer	ACAGACATGGTGCGAAGAGTCTATT
STS R primer	CCGTCCTCCAGGGGGTAGAA

Table S2. Protein marker for immune cell staining

Target		Marker
T cell	CD4+ T cell	CD45+, CD3+, CD4+, CD8-
	CD8+ T cell	CD45+, CD3+, CD8+, CD4-
B cell		CD45+, CD3-, CD19+
NK cell		CD45+, CD3-, CD335+, CD49b+
Macrophage		CD45+, CD11b+, F4/80+, CD11c-
Dendritic cell		CD45+, CD11b+, CD11c+
Marginal-zone B cell (MZB)		CD45+, CD3-, CD19+, CD21+/CD35+, CD23-, CD1d+
Epithelial cell		CD45-, CD326+, CD31-
Endothelial cell		CD45-, CD326-, CD31+

Table S3. Fluorescence information for immune cell staining

Target	Antibody	Fluorescence
T cell	CD45	eFlour506
	CD3	FITC
	CD4	PE
	CD8	Superbright 645
B cell, Marginal-zone B cell (MZB)	CD45	eFlour506
	CD3	FITC
	CD19	PE
	CD21/35	Pacific Blue
	CD23	Violet 605
NK cell	CD45	Superbright 600
	CD3	FITC
	CD335	PE
	CD49b	Pe-cy7
Macrophage, Dendritic cell	CD45	Superbright 600
	CD11b	eFlour506
	F4/80	PE
	CD11c	FITC
Epithelial cell, Endothelial cell	CD45	Superbright 600
	CD326	PE
	CD31	FITC

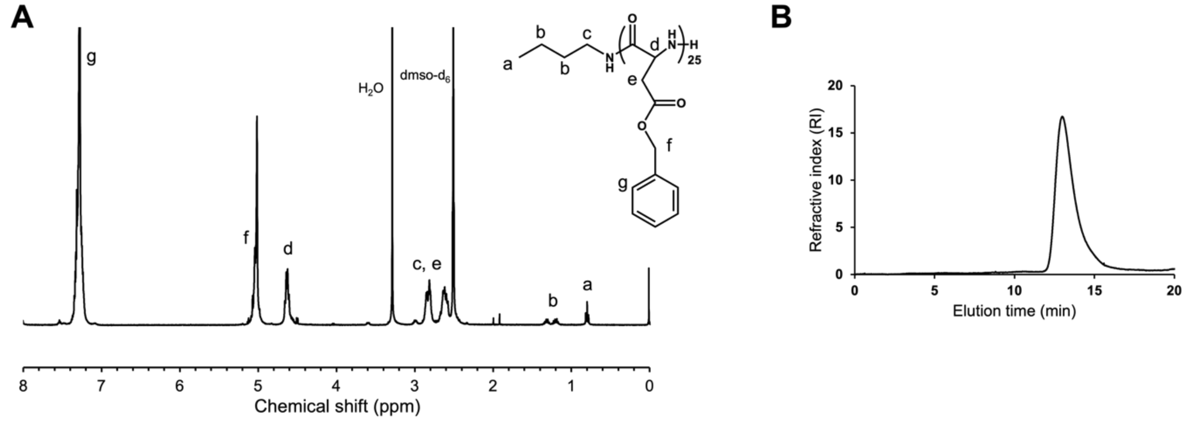

Figure S1. Characterization of PBLA (A) ^{1}H NMR spectrum (400 MHz, 5 mg/mL, DMSO-d_6, 30 °C). (B) SEC chart of PBLA with DP = 25

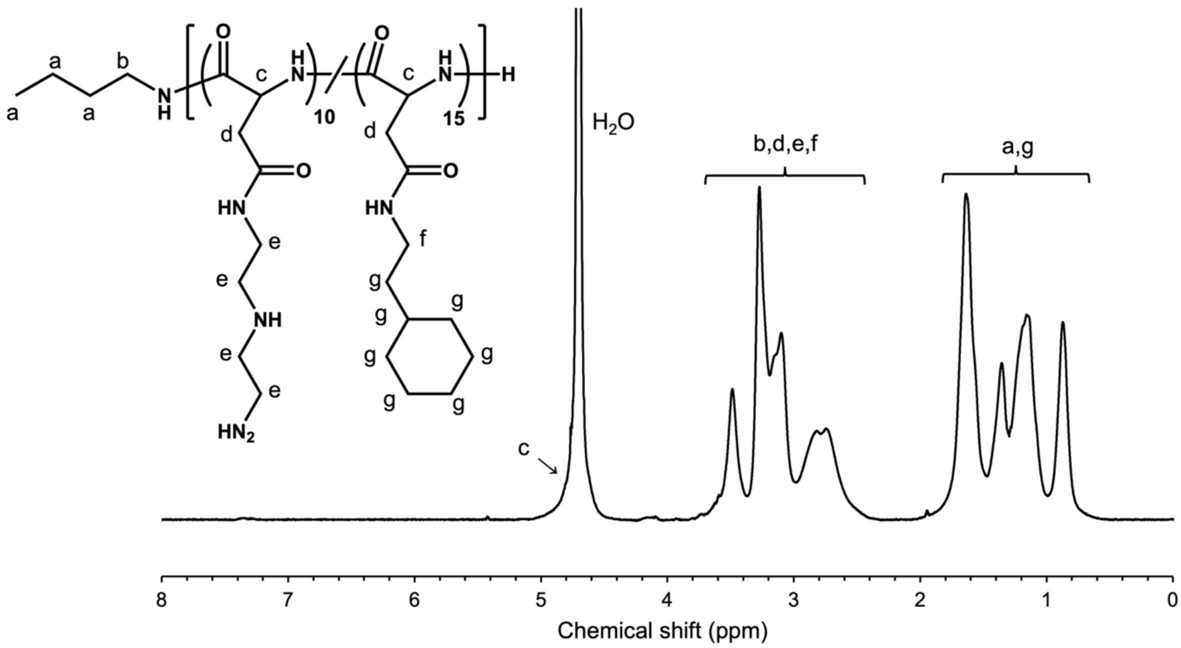

Figure S2. ^{1}H NMR spectrum of PAsp(DET/CHE) (400 MHz, 5 mg/mL, D_2O, 30 °C).

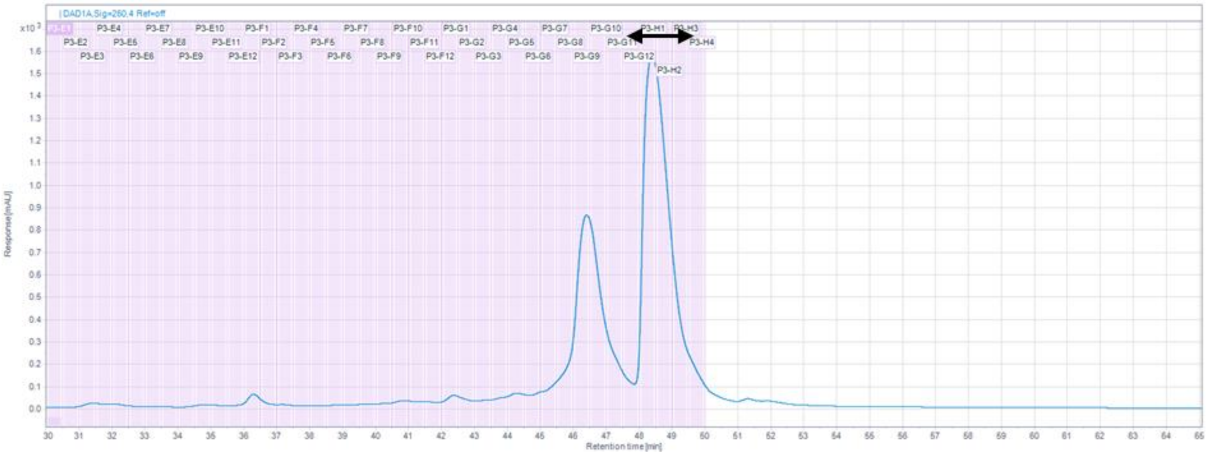

Figure S3. IP-RP HPLC purification of circular RNAs. Arrow indicates peak area which was collected. Then, the eluted circular RNAs were recovered by standard ethanol precipitation method.

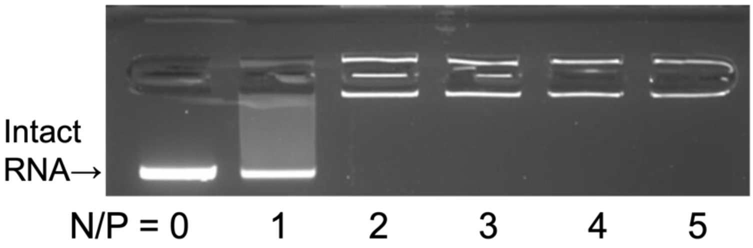

Figure S4. Agarose gel electrophoresis of polyplexes prepared at various N/P ratios between PAsp(DET/CHE) and FLuc circular RNA. The circular RNA on the gel was stained with ethidium bromide.

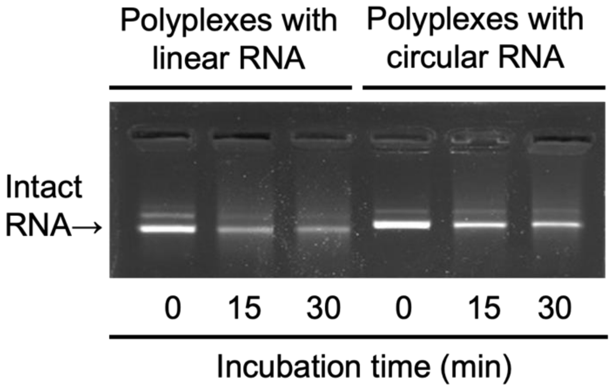

Figure S5. Representative agarose gel electrophoresis of FLuc linear and circular RNA after incubation of polyplex (N/P = 2.8) in 10% FBS at 37 °C. After incubation, polyplex samples were purified with an RNeasy mini kit for RNA release. The linear and circular RNA on the gel was stained with ethidium bromide.

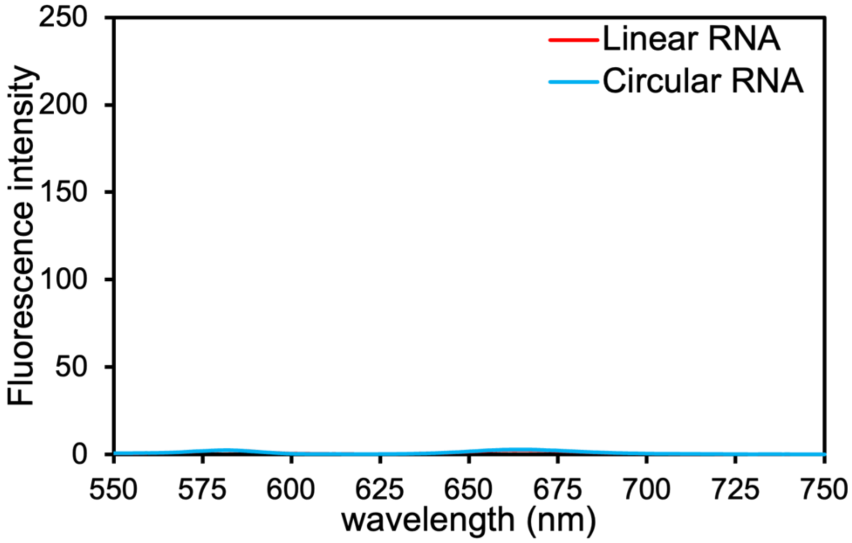

Figure S6. Fluorescence emission profiles of linear and circular Cy5-RNAs only.

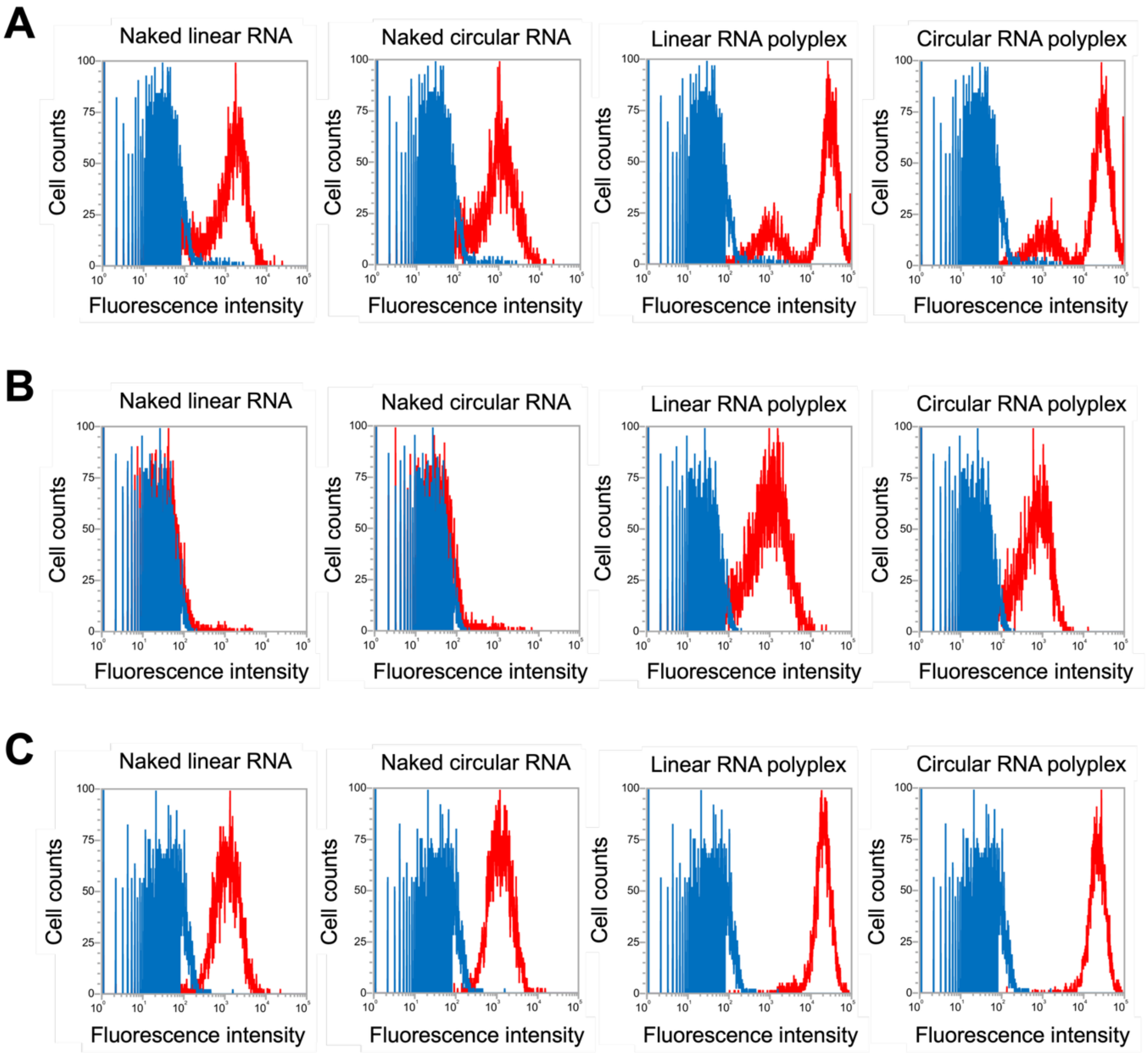

Figure S7. Cellular uptake profiles of naked Cy5-RNA and Cy5-RNA-loaded PAsp(DET/CHE) polyplexes in cultured (A) Jurkat, (B) NK-92mi, and (C) RAW 264.7 in 4-h incubation. Buffer-treated control cells are shown in blue color.

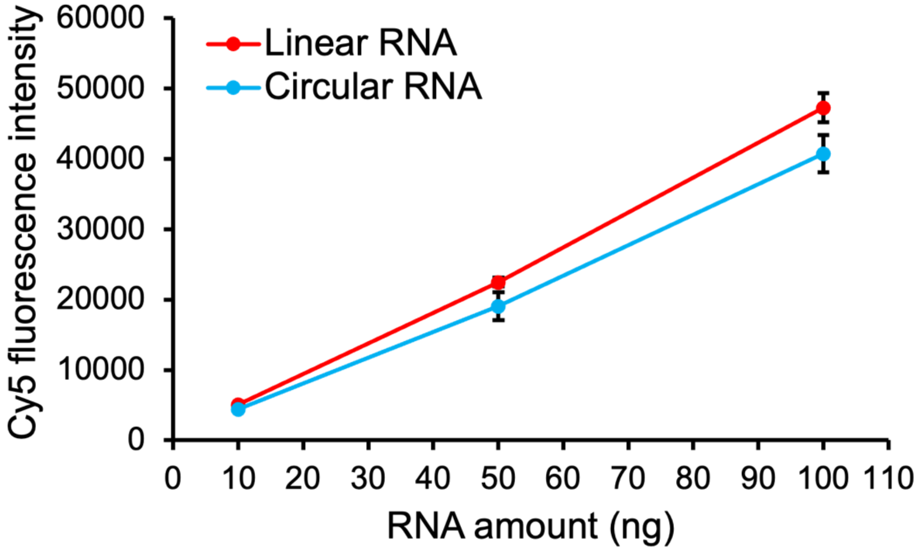

Figure S8. Fluorescence intensities of linear and circular naked Cy5-RNAs, measured by Nanodrop fluorospectrometer.

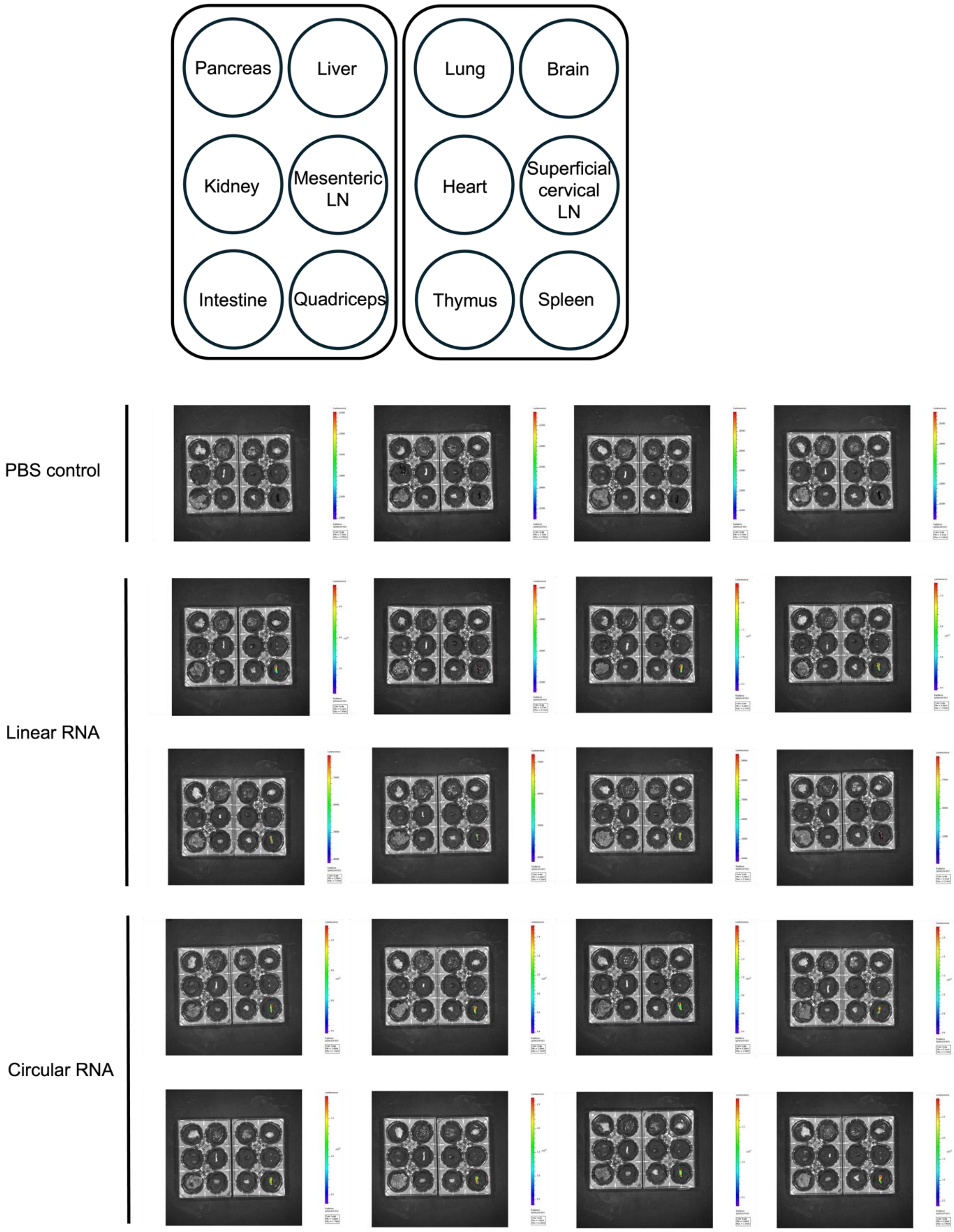

Figure S9. IVIS images of various organs excised from the mice 48 h after intravenous administration.

Table S4. Cell population in spleen in flow cytometric analysis (mean ± SD)

% IN TOTAL SPLEEN CELLS			UNIT: %
Cell types	GROUPS		
	PBS	Linear RNA polyplex	Circular RNA polyplex
CD3+	15.23±1.10	14.57±1.12	14.33±1.67
CD4+ T cell	9.33±0.35	8.95±0.68	9.52±1.09
CD8+ T cell	4.74±0.64	4.60±0.46	4.05±0.62
NK cell	3.80±0.81	3.97±0.85	3.13±0.56
Epithelial	0.00±0.01	0.01±0.01	0.01±0.01
Endothelial	0.23±0.02	0.28±0.04	0.24±0.07
B cell	38.25±2.46	39.28±1.47	36.30±1.96
Follicular B cell	31.80±1.70	32.89±1.36	29.62±1.43
Marginal Zone B	0.89±0.10	1.09±0.17	1.37±0.25
Dendritic cell	0.75±0.06	0.74±0.07	0.64±0.06
Macrophage	0.87±0.20	0.75±0.29	0.84±0.22
n	4	8	8
